# Questioning the radiation limits of life: *Ignicoccus hospitalis* between replication and VBNC

**DOI:** 10.1007/s00203-020-02125-1

**Published:** 2020-12-15

**Authors:** Dagmar Koschnitzki, Ralf Moeller, Stefan Leuko, Bartos Przybyla, Kristina Beblo-Vranesevic, Reinhard Wirth, Harald Huber, Reinhard Rachel, Petra Rettberg

**Affiliations:** 1 Radiation Biology Department, Institute of Aerospace Medicine, German Aerospace Center (DLR e.V.), Linder Hoehe, 51147 Cologne, Germany; 2 Faculty for Biology and Preclinical Medicine, Institute for Microbiology and Archaea Centre, University Regensburg, Regensburg, Germany; 3 Faculty for Biology and Preclinical Medicine, Centre for Electron Microscopy, University of Regensburg, Regensburg, Germany

**Keywords:** *Ignicoccus hospitalis*, Radiation, Survival, Genome integrity, Extremophiles, VBNC

## Abstract

Radiation of ionizing or non-ionizing nature has harmful effects on cellular components like DNA as radiation can compromise its proper integrity. To cope with damages caused by external stimuli including radiation, within living cells, several fast and efficient repair mechanisms have evolved. Previous studies addressing organismic radiation tolerance have shown that radiotolerance is a predominant property among extremophilic microorganisms including (hyper-) thermophilic archaea. The analysis of the ionizing radiation tolerance of the chemolithoautotrophic, obligate anaerobic, hyperthermophilic Crenarchaeon *Ignicoccus hospitalis* showed a *D*_10_-value of 4.7 kGy, fourfold exceeding the doses previously determined for other extremophilic archaea. The genome integrity of *I. hospitalis* after γ-ray exposure in relation to its survival was visualized by RAPD and qPCR. Furthermore, the discrimination between reproduction, and ongoing metabolic activity was possible for the first time indicating that a potential viable but non-culturable (VBNC) state may also account for *I. hospitalis*.

## Introduction

Life as we know, it inhabits our planet Earth since about 3.8 Ga years, and the terrestrial environment and prevailing conditions have changed over time. Several geochemical and isotopic studies demonstrated that the atmosphere during the Archaean Age (approx. 3.8–2.5 Ga ago) was essentially anoxic (Grenfell et al. 2010; Holland 1999). As a consequence, the UV-absorbing ozone layer was absent, enabling short wavelengths of the non-ionizing solar UV radiation spectrum to penetrate into Earth’s surface hence increasing the overall terrestrial UV stress in comparison to today (Cockell and Horneck 2001; Margulis 1976). Early Earth’s ocean was significantly affected by the late heavy bombardment, heaviest until about 3.8 Ga when frequent meteorite impacts may have heated the oceans partially over 100 °C (Nisbet and Sleep 2001). An adaptation to this hot, aqueous environment would have been beneficial for evolution, survival, and successful propagation of life (Miller and Lazcano 1995).

Ionizing radiation, from, e.g., geologic sources by the decay of radioactive elements in Earth’s crust, has severe effects on nucleic acids and genome integrity (Karam et al. 2001). Efficient repair of DNA damage is, therefore, essential for all forms of life (White and Allers 2018). Besides that, a vast variety of different survival strategies by changing biochemical and physiological features have been developed to inhabit a broad range of extreme biological niches (Jung et al. 2017). Focusing here on hyperthermophilic microorganisms, efficient intracellular repair mechanisms (Grogan 1998, 2000) allow them to thrive in their hot habitats which are expected to affect their genome stability by spontaneous decomposition of DNA suggesting that genome replication may depend on DNA repair (Mao and Grogan 2017; Lindahl 1993). Given that DNA damages induced by high temperature are similar to those induced by ionizing radiation, the intrinsic property of hyperthermophiles might be responsible for γ-radiation resistance (Jung et al. 2017) allowing them to withstand periods of high radiation intensities (Beblo et al. 2011). Previous studies addressing the radiation tolerance of different (hyper-) thermophilic archaea including *Ignicoccus hospitalis* demonstrated that radiotolerance is a common property among the tested extremophilic organisms even though a naturally radiation-intensive environment has not been found (Beblo et al. 2011; Jung et al. 2017). The hyperthermophilic crenarchaeon *I. hospitalis*, first described in 2007, was isolated from a submarine hydrothermal system (Paper et al. 2007). The genus *Ignicoccus*, belonging to the order *Desulfurococcales*, represents a deeply branching lineage within the family of *Desulfurococcaceae* (Huber et al. 2000; Huber and Stetter 2001). All members share common morphological and physiological characteristics like an irregular coccoid shape with a cell diameter of 1–4 µm (Huber et al.^2000^, ^2012^), and an optimal growth temperature at 90 °C. They live as chemolithoautotrophic, obligate anaerobes, and grow by reduction of elemental sulfur using hydrogen as electron donor while producing H_2_S (Huber et al. 2000).

The remarkable radiotolerance mentioned above raises the question for the boundaries and capabilities of life as we know it. All previously tested microorganisms prefer an extremophilic lifestyle but are never exposed in their natural habitat to radiation in the kGy range (Beblo et al. 2011). The mechanisms of radiation resistance remain incompletely understood and probably vary between different taxa. They can include non-enzymatic antioxidants like manganese complexes and physiological adjustments to combat oxidative stress (e.g., reduce intracellular free iron concentrations), in addition to efficient DNA repair systems (Shuryak 2019). Fast and efficient DNA repair mechanisms can maintain proper genome integrity under varying harsh environmental conditions, and developing additional survival strategies to withstand unpredictable environmental changes may be advantageous for long-term survival. One prominent bacterial example for this is the so-called viable but non-culturable state (VBNC) of *Escherichia coli* and *Vibrio cholerae* (Xu et al. 1982). Cells entering the VBNC state are alive but no longer able to grow in artificial media, while maintaining their metabolic activity (Oliver 2000). The VBNC state of human bacterial pathogens and its importance has extensively been reviewed by Li et al. (2014), whereas nothing is known in terms of Archaea (Moissl-Eichinger 2011). This lack of knowledge encourages reconsidering the definition of survivability for extremophilic archaea, and was consequently tested with *I. hospitalis* after ionizing radiation treatment. Experimental data presented in this study indicate that a potential VBNC state may also account for this strain, thus (hyperthermophilic) Archaea. A possible discrimination between the ability to replicate, and the metabolic activity may promote a better understanding of the organismic response and tolerance as a consequence thereof.

## Materials and methods

### Strain and culture conditions

The type strain *Ignicoccus hospitalis* KIN4/I^T^ was obtained from the Institute for Microbiology and Archaea Centre, University Regensburg. Cells were cultivated in 120-ml serum bottles containing 20 ml of anoxic, elemental sulfur-containing ½ SME medium (½ SME + S^0^) with a H_2_/CO_2_ (250 kPa; 80:20, *v*/*v*) headspace (Paper et al. 2007). For inoculation, 0.2 ml of a stationary phase culture was used. Incubation was carried out at 90 °C under shaking (60 rpm) for approximately 15 h (final cell concentration around 1 × 10^7^ cells/ml). The cell concentration was determined using a Thoma cell counter chamber (depth: 0.02 mm).

### Ionizing radiation (γ-ray) exposure of *Ignicoccus hospitalis*

Independent irradiation campaigns were performed using the γ-ray source (^60^Co) at BGS (Beta Gamma Service, Wiehl, Germany) with slightly differing doses for technical reasons. The doses applied were either 0, 6.7, 12.7, 19.0, 27.2, 55.8, 81.1, 117.1 kGy or as indicated. Certified dosimetry data were provided by BGS for the respective irradiation campaign.

*I. hospitalis* stationary phase cultures were anoxically exposed to ionizing radiation (0–117.1 kGy) at room temperature. In addition, unexposed laboratory (DLR) and transport control (BGS) samples were kept at room temperature, too. Two milliliters of radiation-exposed and unexposed stationary phase culture was transferred into 20-ml fresh culture medium (1/2 SME + S^0^) followed by serial dilutions to determine the survival. Samples, which were exposed in parallel, were used for genomic DNA extraction. To preclude that the experimental procedure potentially impacts the relative survival of ionizing radiation-exposed cells, *I. hospitalis* cultures were serial diluted (1:10) in ½ SME + S^0^ medium prior to γ-ray exposure (0, 11.6, 17.5 kGy), and directly incubated at 90 °C after irradiation. The surviving fractions were determined by most probable number (MPN) technique (Franson 1985).

### Determination of dose–effect curves after γ-ray exposure based on survival and metabolic activity

**Fig. 1:**
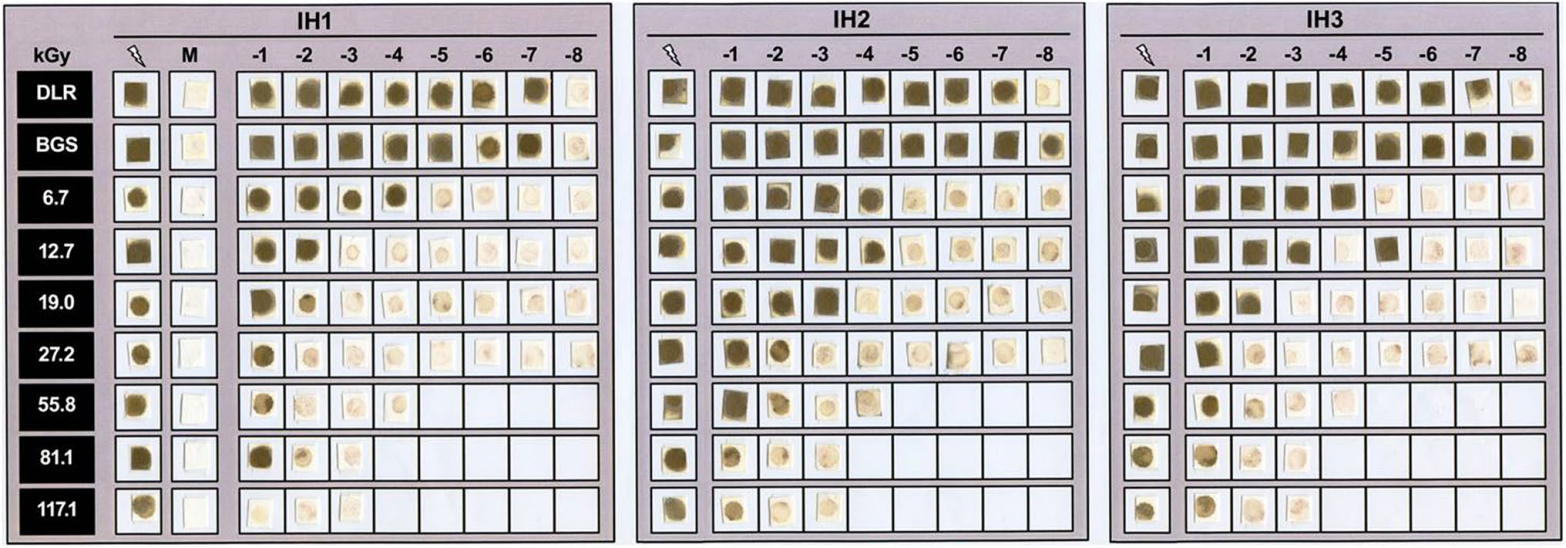
Metabolic activity of *I. hospitalis* (three parallels: IH1, IH2, and IH3) after γ-ray exposure monitored on lead acetate paper. The metabolical produced H_2_S reacts with lead acetate forming lead sulfide, visible as dark-brown spots. *Flash* γ-radiation-exposed cultures, *M* unexposed ½ SME + S^0^ medium incubated at 90 °C for up to 6 days, *DLR* laboratory control, *BGS* transport control. Serial dilutions with 1:10 dilution steps (10^–1^–10^–8^) are represented by the exponent (Koschnitzki 2016)

Ionizing radiation-exposed *I. hospitalis* cells were incubated at 90 °C for up to 6 days to determine the surviving fraction. The metabolic activity (H_2_S production) was qualitatively monitored by dripping 5 µl of the culture onto lead acetate paper (Macherey–Nagel, Düren, Germany). The sulfide ion from metabolically produced H_2_S reacts immediately with lead acetate to insoluble lead sulfide which can be seen as dark-brown spots on the paper (Paper et al. 2007). Three independent experiments were conducted for each dose (Fig. 1). The resulting survival rate was calculated from serial dilutions as relative survival (S = N/N_0_) after exposure to ionizing radiation (N) compared to the untreated laboratory control sample (N_0_). The dose needed to inactivate the population by 90% (*D*_10_) was determined by linear regression from the linear parts of the semi-logarithmically plotted survival curves (Harm 1980).

### Ionizing radiation exposure of ½ SME medium and single-medium components

To investigate the influence of γ-ray-exposed culture medium on the survival of *I. hospitalis*, serum bottles containing 20-ml anoxic ½ SME + S^0^ medium were exposed to increasing ionizing radiation doses (0–117.1 kGy), and used for subsequent serial dilutions of untreated *I. hospitalis* cells. The relative survival of *I. hospitalis* either cultivated in ^60^Co radiation-exposed or unexposed ½ SME medium (½ SME-S^0^) supplemented by exposed or unexposed dry elemental sulfur was determined in an additional approach. Furthermore, small amounts of single substances, needed for ½ SME medium preparations, were exposed to γ-radiation (0, 117.1 kGy) as they were provided by the manufacturer. Dose-specific ½ SME media were prepared from these exposed substances using sterile ddH_2_O, except autoclaving, and either supplemented with dose specific γ-ray-exposed or unexposed elemental sulfur (S^0^). All serum bottles were purged with H_2_/CO_2_ (250 kPa; 80:20, *v*/*v*).

### Extraction of genomic DNA

Genomic DNA was extracted from 20-ml unexposed and ionizing radiation-exposed cells according to the protocol of Tillett and Neilan (2000). The samples were centrifuged at 4 °C, 4500 × g for 1 h, and the resulting pellets resuspended in 700-µl freshly prepared XS-Buffer. The DNA was purified by PCI (25:24:1) purification, isopropanol precipitation, and two washing steps with 70% ethanol. Resulting DNA was resuspended in 30-µl ddH_2_O. DNA preparations from three independently exposed cultures were pooled and used as template for following RAPD-PCR and qPCR analyses. The concentrations were determined by Qubit fluorometric quantitation using Qubit dsDNA HS Assay (Thermo Fischer Scientific, Waltham, MA, USA), and a Qubit 2.0 Fluorometer (Thermo Fischer Scientific, Waltham, MA, USA).

### Determination of the genomic DNA integrity

The genomic DNA integrity after irradiation was analyzed by RAPD-PCR (randomly amplified polymorphic DNA) (Atienzar et al. 2002). Twenty-five nanograms of genomic DNA were used as template. The PCR reaction was carried out in 20 µl containing 1 U Platinum Taq Polymerase (InvitrogenTM, Carlsbad, CA, USA), 1 × PCR buffer, 3.75 mm MgCl_2_, 0.2 mm dNTPs, and 0.5 µm of the primer P2 (5′-GGG GCC CTA C-3′; Lepage et al. 2004). A PeqSTAR 96 Universal cycler (Peqlab, Erlangen, Germany) was used for 40 cycles of amplification (94 °C for 1 min, 42 °C for 1 min, and 72 °C for 2 min) after an initial DNA denaturation step at 94 °C for 10 min. RAPD profiles were subjected to 2% agarose gel electrophoresis in Tris Acetate-EDTA buffer with 7 V/cm for 2.5 h. SYBR Safe (Invitrogen, Carlsbad, CA, USA) was used for staining, and the profiles visualized using an ImageQuant LAS4000 (GE Healthcare, Little Chalfont, UK).

### Determination of the relative amplification rates

Quantitative PCR (qPCR) was used to determine the relative amplification rates for a 1.3 kb amplicon after ionizing radiation exposure. Primers for the 16S rRNA gene sequence were designed to amplify between the position 27 and position 1394 (fwd: 5′-CTAAGCCATGGGAGTCGAAC-3′; rev: 5′-ACGGCTACCTTGTTACGACT-3′) in the genome of *I. hospitalis* (Podar et al. 2008). An Engine Opticon^®^ 2 cycler (CFD-3220, MJ Research Inc., St. Bruno, Canada) was used for 35 cycles of amplification (95 °C for 20 s, 60 °C for 20 s, and 72 °C for 90 s) after an initial DNA denaturation step at 95 °C for 3 min. Five nanograms of genomic DNA were used as template and the amplifications were performed using the KAPA SYBR^®^ FAST 2 × qPCR Master Mix (Peqlab, Erlangen, Germany). The relative amplification rates were calculated according to the normalized *C*_(*t*)_ values by *C*_(*t*)_ normalized the minimum = (Max − value)/(Max − Min) where Max represents the highest and Min the lowest *C*_(*t*)_ value within the experiment, and value is the *C*_(*t*)_ value to be normalized (Hunter et al. 2010). The relative amplification rates were plotted against the applied dose (kGy). To check whether single 16S rRNA amplicons were obtained for every sample, qPCR amplicons were analyzed on a 2% agarose (w/v) gel where 2 µl of sample were loaded per lane.

## Results

### Survival of *I. hospitalis* after gamma radiation exposure

**Fig. 2:**
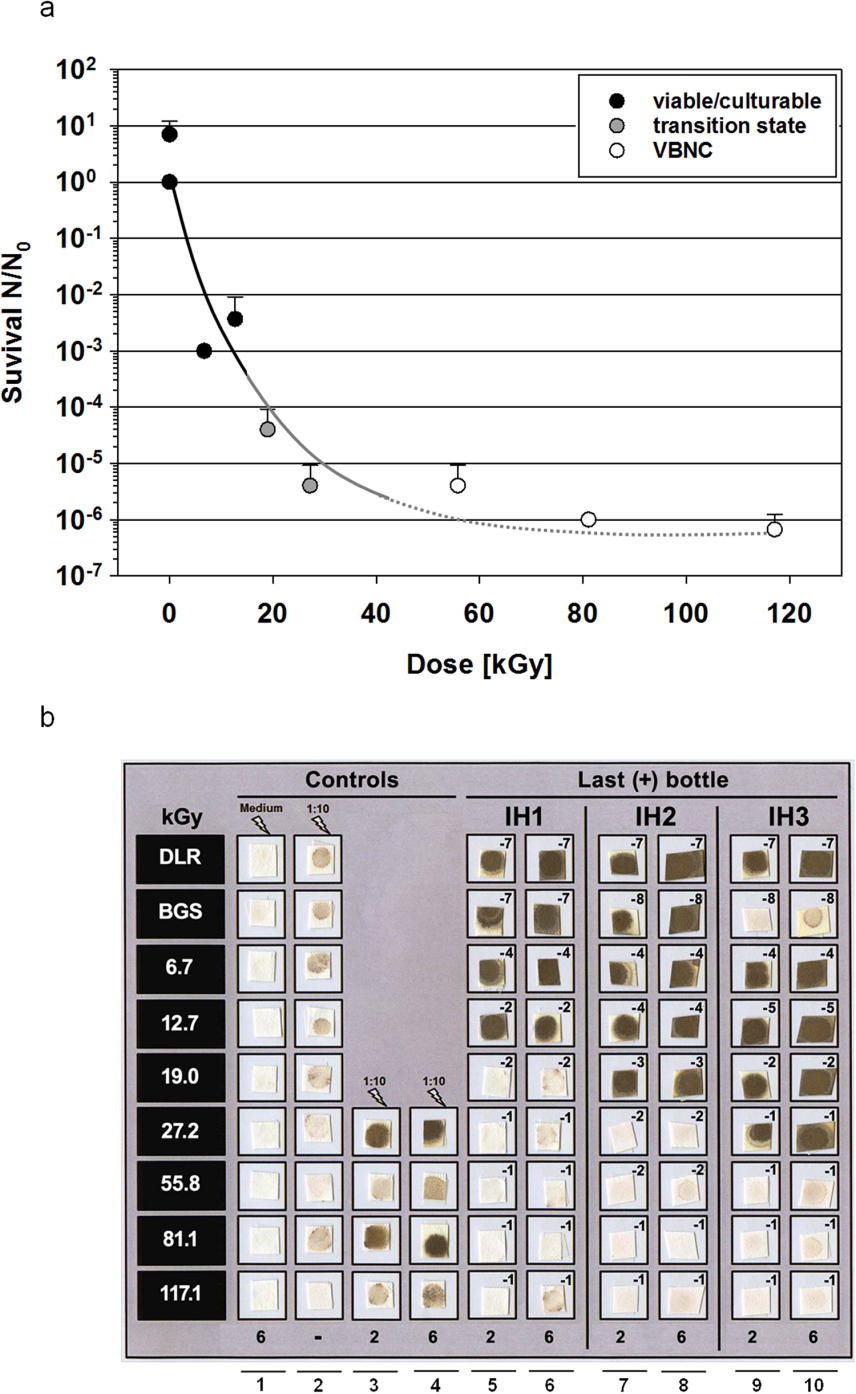
**a** Survival of *I. hospitalis* for reproduction (culturable) and viable but non-culturable (VBNC) state. Black circles: viable and replication-competent, hence culturable. Gray circles: transition state between culturable, and viable but non-culturable (VBNC). Open circle: viable but non-culturable (VBNC). The experiment was conducted with *n* = 3 and refers to the results on lead acetate paper. Trendlines were fitted by hand.** b** Controls to discriminate between the culturable and viable but non-culturable state. Metabolic activity of three independently exposed (γ-radiation) *I. hospitalis* stationary phase cultures (IH1, IH2, and IH3) monitored on lead acetate paper. Exposed ½ SME + S^0^ medium, and exposed stationary phase cultures (1:10 diluted in fresh medium) were incubated at 90 °C for up to 6 days (columns 1, 3, and 4). Exposed IH1 stationary phase cultures were tenfold diluted in fresh medium before incubation (column 2). For higher sensitivity, 0.2 ml of the last positive IH1 bottle within a serial dilution were transferred into fresh medium, and incubated at 90 °C for up to 6 days (columns 5, 6). The same was conducted with IH2 (columns 7, 8), and IH3 (columns 9, 10). *DLR* laboratory control, *BGS* transport control. Serial dilutions with 1:10 dilution steps (10^–1^–10^–8^) are represented by the exponent (− 1 to − 8), and 2 and 6 refer to the days of incubation (Koschnitzki 2016)

The survival of *I. hospitalis* after ionizing radiation exposure was determined. Ongoing metabolic activity, here H_2_S production qualitatively monitored on lead acetate paper, was detected for cells exposed to > 55.8 kGy (Fig. 1). As a control, unexposed ½ SME + S^0^ medium, incubated at 90 °C for 6 days, did not result in any positive signal. All signals obtained within the serial dilutions were assessed as positive signals due to organismic, metabolic activity. Thus, ionizing radiation exposure of *I. hospitalis* to ~ 25 kGy resulted in an inactivation by five orders of magnitude, allowing to determine a *D*_10_-value of 4.7 kGy (Fig. 2a). A comparable *D*_10_-value was determined by Beblo et al. (2011), supporting this high radiation tolerance of *I. hospitalis*.

### Discrimination between culturability and VBNC

To demonstrate a potential VBNC state in the domain of Archaea (Fig. 2), different control samples were analyzed to exclude autonomous H_2_S-production by chemical or thermal reactions in the absence of *I. hospitalis* cells which might result in false-positive signals on lead acetate paper after ionizing radiation exposure. However, no H_2_S was produced (no lead sulfide was detected) in irradiated ½ SME + S^0^ medium incubated at 90 °C (Fig. 2b; column Medium). Therefore, all positive signals obtained were assumed as organismic, metabolic activity. A tenfold dilution of ionizing radiation-exposed *I. hospitalis* stationary phase cultures in fresh ½ SME + S^0^ medium resulted in only light-brown spots before incubation; this signal can be ascribed to dissolved H_2_S which was metabolically produced prior to exposure (Fig. 2b; column 1:10). *I. hospitalis* cells which were exposed to doses > 27.2 kGy and tenfold diluted before incubation showed positive signals on lead acetate paper supporting the results presented in Fig. 1. To further increase sensitivity, 20 ml of ½ SME + S^0^ medium was inoculated with only 0.2 ml from the serum bottle giving the last positive lead acetate paper signal within the serial dilution (see Fig. 1), and was repeated for all independently exposed samples (IH1, IH2, IH3; Figs. 1, 2b). As a result, a dose < 19.0 kGy decreased the survival by around three orders of magnitude, and is seen as viable/culturable state (Fig. 2; filled circles). Metabolic activity (hence active H_2_S production) was visualized on lead acetate paper (Fig. 2b). An applied dose ranging between 19.0 and 27.2 kGy (Fig. 2b; IH2, IH3), represented by gray circles in Fig. 2a, is defined as transition state. Within these three independently exposed *I. hospitalis* samples, only two (Fig. 2b; IH2, IH3) gave positive signals on lead acetate paper when 100-fold diluted (0.2 ml in 20 ml ½ SME + S^0^ medium). The ability of reproduction/cell division ended with an applied dose of > 27.2 kGy (Fig. 2a; open circles), although metabolic activity was maintained. The ability of cell division was forfeited resulting in a potential VBNC state (Fig. 2a). Positive signals due to H_2_S production after exposure to even higher doses can be seen in Fig. 1, when exposed *I. hospitalis* cells were tenfold diluted. The same samples did not give any positive signal when 100-fold diluted (Fig. 2b). The concentration of cells did not increase after 6 days of incubation due to the loss of the ability to divide. Thus, the concentration of metabolically active *I. hospitalis* cells producing H_2_S was too low to form insoluble lead sulfide.

### Influence of the surrounding medium on the survivability of *I. hospitalis*

**Fig. 3:**
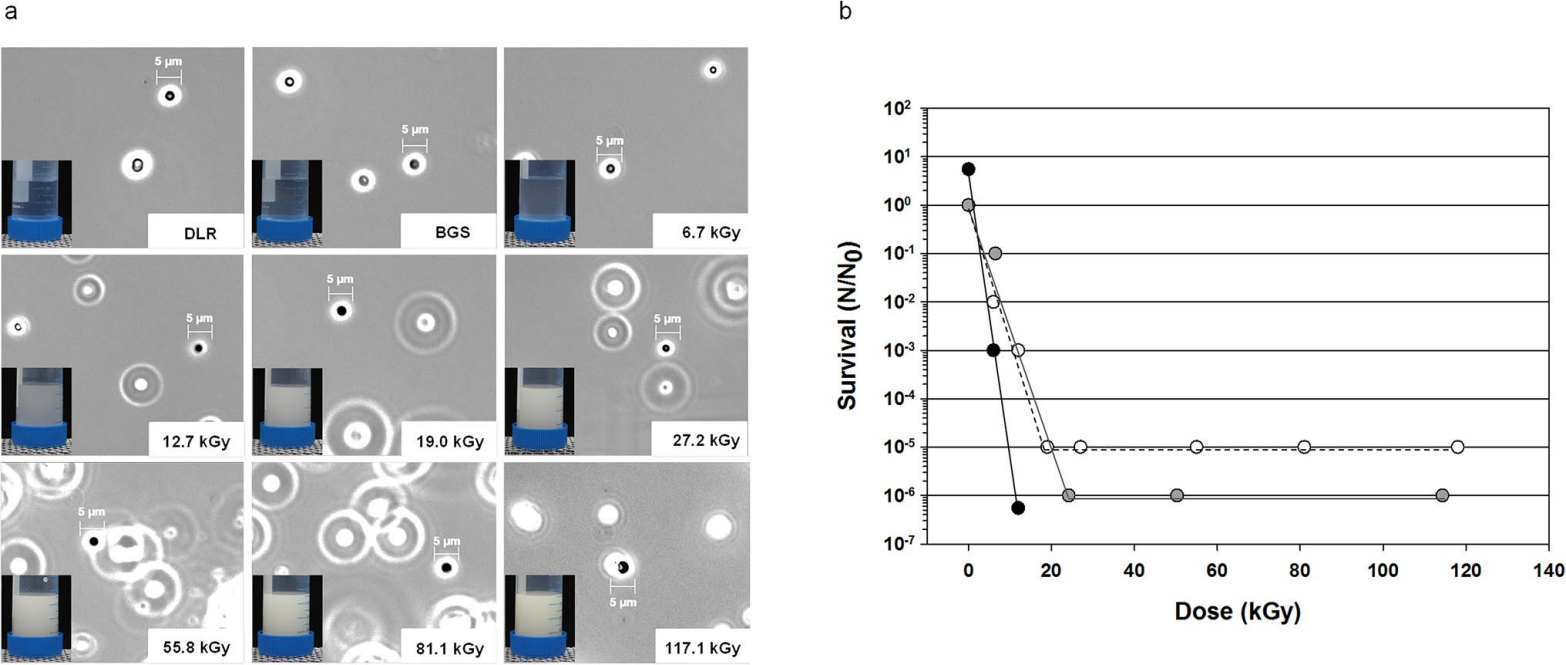
**a** Anoxic exposure of *I. hospitalis* stationary phase cultures to gamma radiation, and transfer into Falcon^®^ tubes after irradiation. *I. hospitalis* cells can be seen as black cocci (scale bar = 5 µm), whereas strong refractive particles are ascribed to elemental sulfur. The numbers in the lower right corner represent the applied dose in kGy. DLR represents the laboratory control, whereas BGS stands for the transport control. **b** Influence of γ-ray-exposed, sulfur-containing and sulfur-free ½ SME medium on the survival of *I. hospitalis* in comparison to serial diluted γ-ray-exposed *I. hospitalis* cells. Black circles: serial dilution of *I. hospitalis* stationary phase cells prior to exposure. The diluted samples were exposed to ionizing radiation, and directly incubated afterwards. Open circles: serial dilution of untreated *I. hospitalis* cells in γ-ray-exposed ½ SME + S^0^ medium. Gray circles: serial dilution of untreated *I. hospitalis* cells in γ-ray-exposed ½ SME-S^0^ medium supplemented with unexposed S^0^ after exposure. The experiments were conducted with *n* = 1 and the trendlines fitted by hand (Koschnitzki 2016)

Ionizing radiation is known to be a source of free radical (ROS) formation, produced via the radiolysis of aqueous solutions (Kottemann et al. 2005). We, therefore, tested the influence of ionizing radiation on the culture medium itself (i.e., the environment of the organism), and we detected an increasing turbidity of the surrounding medium correlating with an increasing ionizing radiation dose applied (Fig. 3a, Falcon^®^ tubes). *I. hospitalis* cells appeared as dark cocci with a diameter of around 3 µm independent of the doses applied (Fig. 3a). The increasing amount of strong refractive particles, ascribed to elemental sulfur particles, correlated with increasing turbidity, too (Fig. 3a). Furthermore, *I. hospitalis* cells showed reduced tolerance to γ-radiation when serial diluted in ½ SME + S^0^ medium prior to exposure (Fig. 3b; black circles) compared to cells which were exposed to similar ionizing radiation doses, but serial diluted into ½ SME + S^0^ medium after exposure (Fig. 1). To further investigate whether the exposure of the cultivation medium itself has a negative or inhibitory effect on cell survival, ½ SME + S^0^ medium was exposed to γ-rays, and used for serial dilutions with untreated cells (Fig. 3b; open circles). Gamma radiation-exposed ½ SME + S^0^ medium has an inhibitory effect on cell survivability and can be compared to the results obtained for *I. hospitalis* cultures, which were serial diluted prior exposure. An exposure to ~ 20 kGy reduced the survival by around five orders of magnitude, but the log reduction of cells serial diluted in gamma ray-exposed ½ SME + S^0^ medium did not decrease with increasing dose (> 20 kGy) (Fig. 3b; open circles). The *D*_10_-value for cells serial diluted prior to exposure was around 2.5 kGy, whereas ~ 3.5 kGy was needed to inactivate the population when serial diluted in exposed ½ SME + S^0^ medium). In addition, sulfur-free γ-ray-exposed ½ SME medium (Fig. 3b; gray circles), which was supplemented with unexposed S^0^ after γ-ray exposure, shows a similar inactivation tendency on untreated *I. hospitalis* cells compared to radiation-exposed sulfur-containing ½ SME (Fig. 3b; open circles).

**Fig. 4:**
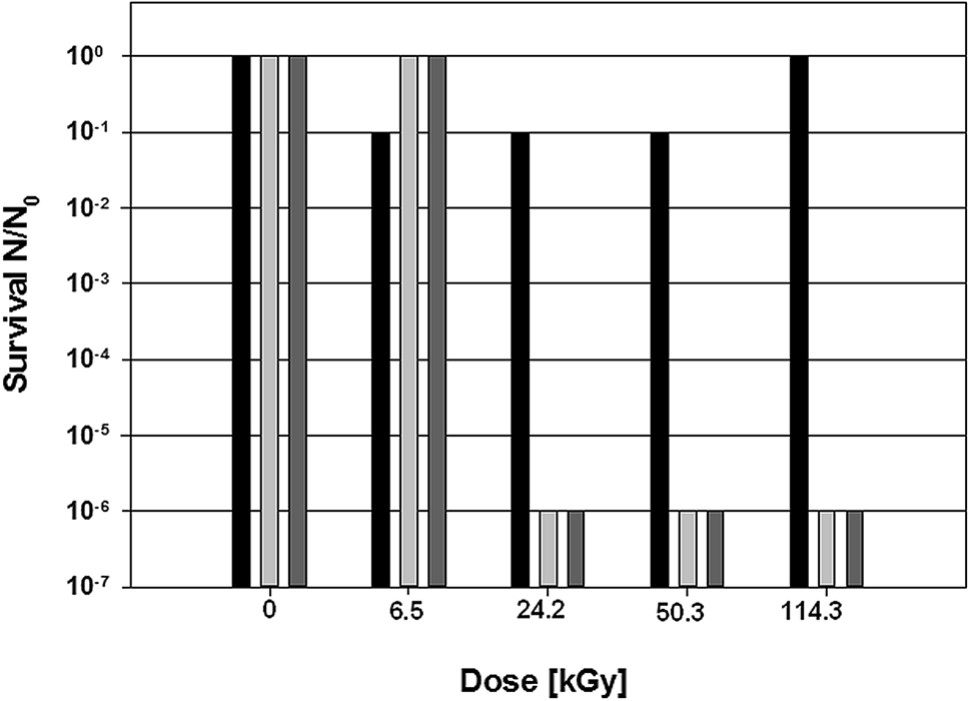
Survival of *I. hospitalis* cells either cultivated in ^60^Co radiation-exposed or unexposed ½ SME medium supplemented with exposed or unexposed elemental sulfur (N). N_0_ represents *I. hospitalis* cells cultivated in unexposed ½ SME + S^0^ medium. Black bar: ½ SME medium (unexposed) + sulfur (exposed). Light-gray bar: ½ SME medium (exposed) + sulfur (unexposed). Dark-gray bar: ½ SME medium (exposed) + sulfur (exposed). Non-linear scale for ease of display. The experiment was conducted with *n* = 1 (Koschnitzki 2016)

The medium composition itself seems to have a negative effect on the survival of *I. hospitalis* after γ-radiation exposure. ½ SME medium without elemental sulfur (½ SME-S^0^) was exposed to γ-radiation and unexposed (Fig. 4; light-gray bars) or exposed (dark-gray bars) dry elemental sulfur S^0^ was added after treatment. An inhibitory effect on cell survival, in the range of six orders of magnitude, was observed in both cases compared to unexposed ½ SME-S^0^ with y-ray-exposed S^0^ (black bars).

Furthermore, we tried to mimic the inhibitory effect of radiation-exposed medium by irradiation of single substances, and subsequent dose-specific medium preparation. A strong color change caused by high-energy ionizing radiation exposure was observed in all halide compounds (NaCl, NaBr, and KI), NaHCO_3_ and Na_2_S × 9 H_2_O (data not shown). The solubility of the single substances in water was not affected and no inhibitory effect on cellular survivability was observed in ½ SME medium prepared from these substances regardless of the supplied sulfur variant (± γ-radiation exposure; data not shown).

### Genomic DNA integrity and relative amplification rates after γ-radiation exposure

**Fig. 5:**
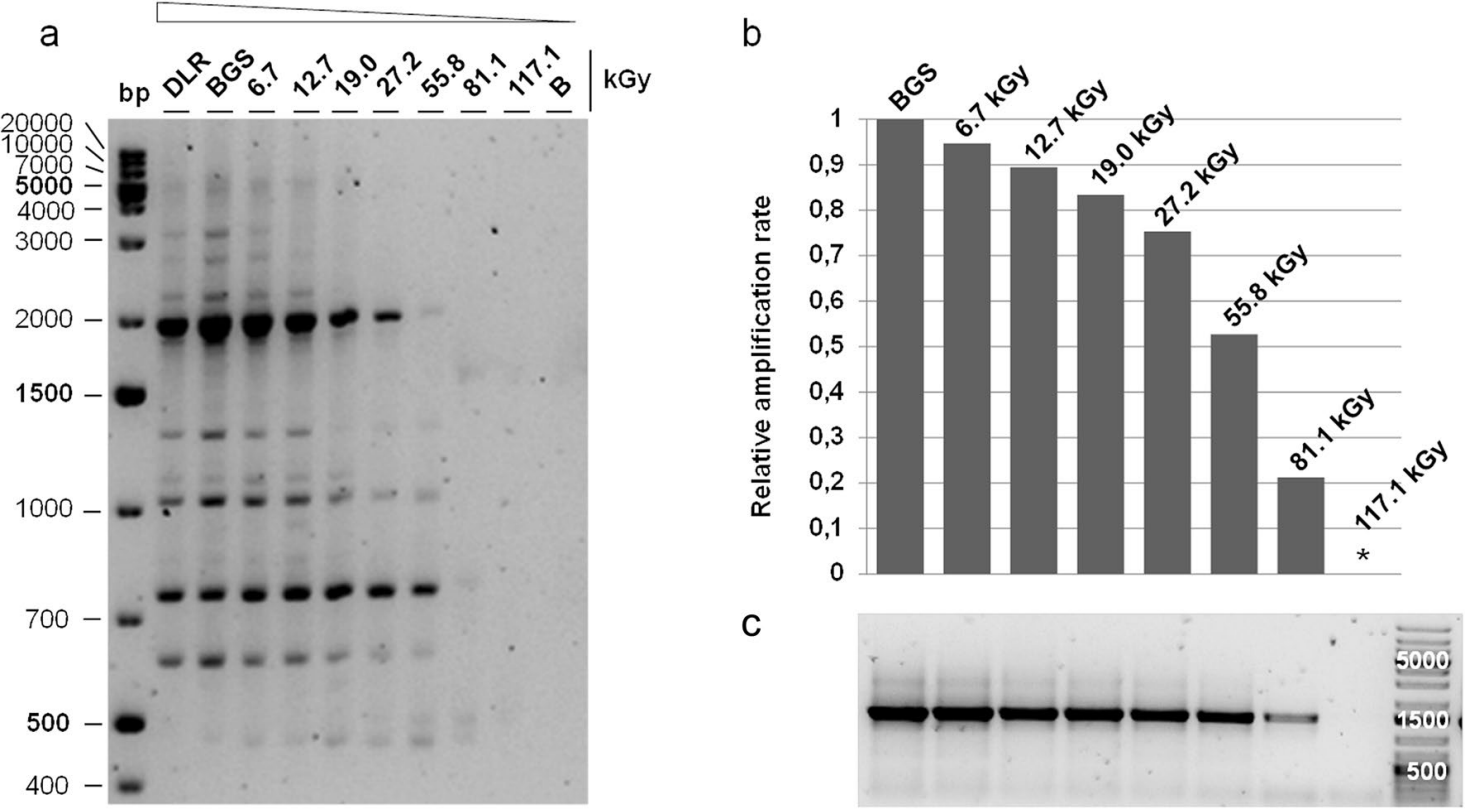
**a** Analysis of the genomic DNA integrity of *I. hospitalis* cells after γ-ray exposure. The RAPD profile was analyzed on a 2% agarose gel. The numbers indicate the applied dose. The arrow indicates decreasing genomic DNA integrity with increasing ionizing radiation dose applied. **b** qPCR with 16S rRNA primer to determine the relative amplification rate after γ-ray exposure. The *C*_(*t*)_ values were normalized the minimum with DLR (laboratory control) acting as untreated reference sample. qPCR analysis was conducted with *n* = 1. **c** Primer specific qPCR amplicon analyzed on a 2% agarose gel, 2 µl were loaded per lane. *No signal, *DLR* laboratory control, *BGS* transport control, *B* blank sample (Koschnitzki 2016)

The RAPD band pattern analysis of *I. hospitalis* revealed that genomic DNA was severely impacted by ionizing radiation compared to the untreated control samples (DLR, BGS; Fig. 5a); the loss of bands increased with increasing doses indicating a loss of potential primer binding sites within the genomic DNA template. An exposure to 19.0 kGy resulted in the absence of the bands with a length of > 2000 bp, and 1400 bp (Fig. 5a). A supportive result was obtained by qPCR. The underlying principle of this method is to measure the integrity of genomic DNA after ionizing radiation treatment by amplifying a long DNA target without assessing the specific nature of the lesion (Furda et al. 2014). The DNA damage to be detected is thereby influenced by the length of the amplicon, with a long amplicon being desired (Ayala-Torres et al. 2000; Leuko and Rettberg 2017). This qPCR method relies on the ability of specific DNA lesions to block the progression of the polymerase on the template strand with the result that gene-specific damage can be measured as decreased amplification of the gene of interest (Ayala-Torres et al. 2000; Leuko and Rettberg 2017). The specific 16S rRNA primer set, was used to amplify a 1.3 kb fragment of the 16S rRNA sequence from genomic DNA. The relative amplification rates were calculated according to the normalized *C*_*t*_ values. As a result, the overall amplification rate decreased with increasing radiation dose compared to the untreated control sample (BGS; Fig. 5b); the agarose gel of the qPCR amplified 16S rRNA fragments emphasized this result (Fig. 5c), showing single 16S rRNA amplicons of around 1.3 kb for every respective sample. An exposure to 117.1 kGy prevented amplification (Fig. 5c).

## Discussion

Radiation tolerance can be found in all three domains of life (Bacteria, Archaea, Eukarya) (Jung et al. 2017). Extreme radiation tolerance, however, predominates the prokaryotic lineage, and can be found in the domain Bacteria (Daly 2009) as well as Archaea (Beblo et al. 2011). For an efficient proteome and genome protection, distinct molecular mechanisms exist in these microorganisms to resist radiation of non- and ionizing nature and other toxic agents (Krisko and Radman 2013). One prominent bacterial example for an extremely radiotolerant microorganism is *Deinococcus radiodurans*. This representative is the most radioresistant bacterium known so far with a *D*_10_-value of 10 kGy (Daly 2009). Several other Bacteria and Archaea, including extremophilic ones, like the hyperthermophilic archaea *Archaeoglobus fulgidus* or *Pyrococcus furiosus* have been investigated in respect to their radiotolerance as well. Both showed a comparable high *D*_10_-value of around 1 kGy (Beblo et al. 2011) in comparison to the mesophilic bacterium *Escherichia coli* (*D*_10_-value 0.25 kGy; Clavero et al. 1994).

Bacterial viability but non-culturability and the reversal of this status (resuscitation) has already been known since three decades (Whitesides and Oliver 1997); however, nothing is known in terms of Archaea (Moissl-Eichinger 2011). The underlying results strikingly demonstrate the remarkable radiation tolerance of *Igni*c*occus hospitalis*, and its ongoing metabolic activity. We were able to discriminate between the survival in terms of reproduction and its metabolic activity after exposure to extremely high doses of γ-radiation. This phenomenon allowed, for the first time, the postulation of a potential VBNC state in the domain of Archaea, and supports this hypothesis empirically by experimentation. This phenomenon raises the question for the boundaries and capabilities of life as we know it, and the accompanying organismic response to external stressors (Moeller et al. 2017). A “dead cell” was long assumed as a cell being unable to multiply, but Lleò and colleagues considered this expression as insufficient (Lleò et al. 2000). They redefined this expression as “a cell being unable to express genes and/or the loss of a cell’s ability to return to the culturable state”, respectively. Here, the VBNC state of *I. hospitalis* allows speculating that this state does also exist in other hyperthermophilic archaea. Different conditions potentially inducing resuscitation need to be tested. Further investigation should test whether the production of mRNA molecules can also be monitored during this process as shown by Lleò and colleagues for *Enterococcus faecalis* (Lleò et al. 2000). The discrimination between the ability of reproduction and metabolic activity helps us to better understand the organismic tolerance and overall response to given stressors.

Besides optimal organismic adaptation to the natural habitat, the propagation of life in an unfavorable environment benefits from cellular responses that may also be advantageous during additional unpredictable stress exposure. The results presented here demonstrate that the environment itself (the corresponding culture medium) plays a role in radiation tolerance and cell survivability. Both, exposed ½ SME + S^0^ and ½ SME-S^0^ medium (S^0^ added after radiation treatment), showed comparable inhibitory effects on cell survivability. This may indicate that the composition of the medium can undergo unfavorable changes resulting in negative effects impacting the cellular survival after ionizing radiation exposure. Saran and Bors discussed in 1997 that cells suspended in physiological saline (PBS) experience varying concentrations of hydrogen peroxide (H_2_O_2_), hypochlorite (HOCl) and the hypochlorite radical anions which are formed in this buffer during irradiation. The chemical half-life of H_2_O_2_ and HOCl during this process is in the order of seconds, but they proposed that this may be enough time to damage the cells substantially (Saran and Bors 1997). The production of these cytotoxic agents may also occur during irradiation of ½ SME medium (+ S^0^/ − S^0^) due to high amounts of NaCl, KCl and KH_2_PO_4_, substances used also for PBS preparation. Due to their short half-lives, other compounds than H_2_O_2_ and HOCl may be responsible for the observed inhibitory effect of radiation-exposed ½ SME + S^0^ medium. A reduced or altered bioavailability of a substance needed for proper metabolism (here elemental sulfur) has to be taken into account, too. It has been observed that elemental sulfur seems to undergo a conformational change upon γ-radiation exposure, seen by increasing turbidity with increasing radiation dose (Fig. 3a). The environment and available energy sources may undergo unfavorable changes upon external impacts, here ionizing radiation exposure, leading to an additional stress for microbial life and its potential propagation.

Reliable cellular responses to altered environmental conditions are a key to success for evolution and propagation of life. Radiation, especially ionizing radiation has adverse effects on organismic genome integrity (Baumstark-Khan and Facius 2001). Here, the genomic DNA integrity of *I. hospitalis* was analyzed after gamma radiation exposure using two different PCR-based applications. The random amplified polymorphic DNA (RAPD) method uses one single primer of arbitrary nucleotide sequence (here: decamer), able to anneal to multiple regions of the genomic DNA (Atienzar et al. 2002). The amplification reaction generates a number of amplicons of variable length which can be visualized by gel electrophoresis. The changes in the genomic DNA, occurring upon different damaging treatments (here: gamma radiation exposure), can be seen in the specific RAPD profile by discrete DNA products like gain/loss of bands and band intensity. These qualitative results can be compared to untreated samples giving an idea of the alterations variance after treatment (Kumar and Gurusubramanian 2011; Atienzar et al. 2002). An increasing amount of DNA lesions in a dose-dependent manner was shown, and exposure to 117.1 kGy prevented amplification (Fig. 5b, c). An altered RAPD pattern, here loss of bands, with increasing radiation intensity (Fig. 5 a) supports this result. Comparable results were obtained for the halophilic archaea *Halobacterium salinarum* NRC-1, *Halococcus morrhuae*, and *Halococcus hamelinensis.* A similar increasing amount of DNA lesions in a dose-dependent manner was shown resulting in a 100% lesion occurrence after 112 kGy (Leuko and Rettberg 2017).

No predictions concerning unspecific changes in other targets like proteins can be made by both PCR-based methods (qPCR, RAPD). Comparing the enormous impact of ionizing radiation in the kGy range on genome integrity, it is highly surprising that *I. hospitalis* is able to survive (here: able to reproduce) doses of up to ~ 19 kGy. An imaginable explanation for this phenomenon would be polyploidy, an increased genome copy number, seen for, e.g., the radiotolerant Euryarchaeon *H. salinarum* which may result in an enhanced resistance against DNA-damaging conditions inducing dsDNA breaks (Kottemann et al. 2005). Besides *H. salinarum*, the highly radiation- and desiccation-tolerant *D. radiodurans* (Mattimore and Battista 1996) shows varying haploid genome copies during exponential or stationary growth phase (Makarova et al. 2001). Gene redundancy may allow the mutation of the genome under unfavored conditions keeping the wild-type information in another copy (Hildenbrand et al. 2011). Polyploidy is often found in euryarchaeal species potentially offering evolutionary advantages (Spaans et al. 2015; Hildenbrand et al. 2011), and possibly also for Crenarchaeota although not yet determined (Hildenbrand et al. 2011; Bernander and Poplawski 1997; Lundgren et al. 2008). A potentially increased copy number may, therefore, correlate with the demonstrated extremely high radiation tolerance (Beblo et al. 2009), but was not yet demonstrated for *Ignicoccus hospitalis*.

The molecular evolution analysis indicates that anaerobic sulfur-reducing chemotrophic hyperthermophiles may act as the oldest recognizable prokaryotes (according to Pace 1991; according to Miller and Lazcano 1995), and an overall adaptation to a hot environment would have been beneficial to survive the last ocean-boiling asteroid impact around 3.8 Ga ago independent from the environmental origin. (Hyper-) thermophilic organisms including *Ignicoccus hospitalis* are known to be highly radiotolerant (Beblo et al. 2011), and seem to be the most suitable survivors after this late heavy bombardment, indicating them at least as potential candidates for early Earth inhabitants (Miller and Lazcano 1995).

## References

[CR1] Atienzar FA, Venier P, Jha AN, Depledge MH (2002). Evaluation of the random amplified polymorphic DNA (RAPD) assay for the detection of DNA damage and mutations. Mutat Res.

[CR2] Ayala-Torres S, Chen Y, Svoboda T, Rosenblatt J, Van Houten B (2000). Analysis of gene-specific DNA damage and repair using quantitative polymerase chain reaction. Methods.

[CR3] Baumstark-Khan C, Facius R, Horneck G, Baumstark-Khan C (2001). Life under conditions of ionizing radiation. Astrobiology: the quest for the conditions of life.

[CR4] Beblo K, Rabbow E, Rachel R, Huber H, Rettberg P (2009). Tolerance of thermophilic and hyperthermophilic microorganisms to desiccation. Extremophiles.

[CR5] Beblo K, Douki T, Schmalz G, Rachel R, Wirth R, Huber H (2011). Survival of thermophilic and hyperthermophilic microorganisms after exposure to UV-C, ionizing radiation and desiccation. Arch Microbiol.

[CR6] Bernander R, Poplawski A (1997). Cell cycle of the thermophilic archaea. J Bacteriol.

[CR7] Clavero MRS, Monk JD, Beuchat LR, Doyle MP, Brackett RE (1994). Inactivation of *Escherichia coli* O157:H7, *Salmonellae*, and *Campylobacter jejuni* in raw ground beef by gamma irradiation. Appl Environ Microbiol.

[CR8] Cockell CS, Horneck G (2001). The history of the UV radiation climate of the earth- theoretical space-based observations. Photochem Photobiol.

[CR9] Daly MJ (2009). A new perspective on radiation resistance based on *Deinococcus radiodurans*. Nat Rev Microbiol.

[CR10] Franson MAH (1985). Standard methods for the examination of water and wastewater.

[CR11] Furda A, Santos JH, Meyer JN, Van Houten B (2014). Quantitative PCR-based measurement of nuclear and mitochondrial DNA damage and repair in mammalian cells. Methods Mol Biol.

[CR12] Grenfell JL, Rauer H, Selsis F, Kaltenegger L, Beichman C, Danchi W (2010). Co-evolution of atmosphere, life, and climate. Astrobiology.

[CR13] Grogan DW (1998). Hyperthermophiles and the problem of DNA stability. Mol Microbiol.

[CR14] Grogan DW (2000). The question of DNA repair in hyperthermophilic archaea. Trends Microbiol.

[CR15] Harm W (1980). Biological effects of ultraviolet radiation.

[CR16] Hildenbrand C, Stock T, Lange C, Rother M, Soppa J (2011). Genome copy numbers and gene conversion in methanogenic archaea. J Bacteriol.

[CR17] Holland HD (1999). When did the Earth’s atmosphere become oxic? A reply. Geochem News.

[CR18] Huber H, Stetter KO (2001) Order II. Desulfurococcales ord. nov. In: Garrity G, Boone DR, Castenholz RW (eds) Bergey’s manual of systematic bacteriology, 2nd edn, vol 1. Springer, New York, pp 179–180

[CR19] Huber H, Burggraf S, Mayer T, Wyschkony I, Rachel R, Stetter KO (2000). *Ignicoccus* gen. nov., a novel genus of hyperthermophilic, chemolithoautotrophic Archaea, represented by two new species, *Ignicoccus islandicus* sp. nov. and *Ignicoccus pacificus* sp. nov. Int J Syst Evol Microbiol.

[CR20] Huber H, Küper U, Daxer S, Rachel R (2012). The unusual cell biology of the hyperthermophilic Crenarchaeon *Ignicoccus hospitalis*. Antonie Van Leeuwenhoek.

[CR21] Hunter SE, Jung D, Di Giulio RT, Meyer JN (2010). The QPCR assay for analysis of mitochondrial DNA damage, repair, and relative copy number. Methods.

[CR22] Jung K-W, Lim S, Bahn Y-S (2017). Microbial radiation-resistance mechanisms. J Microbiol.

[CR23] Karam PA, Leslie SA, Anbar A (2001). The effects of changing atmospheric oxygen concentrations and background radiation levels on radiogenic DNA damage rates. Health Phys Soc.

[CR24] Koschnitzki D (2016) The radiation tolerance of *Ignicoccus* species - their astrobiological relevance and implications to DNA repair processes. Dissertation, University Regensburg

[CR25] Kottemann M, Kish A, Iloanusi C, Bjork S, DiRuggiereo J (2005). Physiological responses of the halophilic archaeon *Halobacterium* sp. strain NRC1 to desiccation and gamma irradiation. Extremophiles.

[CR26] Krisko A, Radman M (2017). Biology of extreme radiation resistance: the way of Deinococcus radiodurans. Cold Spring Harb Perspect Biol.

[CR27] Kumar NS, Gurusubramanian G (2011). Random amplified polymorphic DNA (RAPD) markers and its applications. Sci Vis.

[CR52] Lepage E, Marguet E, Geslin C, Matte-Tailliez O, Zillig W, Forterre P, Tailliez P (2004). Molecular diversity of new thermococcales isolates from a single area of hydrothermal deep-sea vents as revealed by randomly amplified polymorphic DNA fingerprinting and 16S rRNA gene sequence analysis. Applied and Environmental Microbiology.

[CR28] Leuko S, Rettberg P (2017). The effects of HZE particles, γ and X-ray radiation on the survival and genetic integrity of *Halobacterium salinarum* NRC-1, *Halococcus hamelinensis*, and *Halococcus morrhuae*. Astrobiology.

[CR29] Li L, Mendis N, Trigui H, Oliver JD, Faucher SP (2014). The importance of the viable but non-culturable state in human bacterial pathogens. Front Microbiol.

[CR30] Lindahl T (1993). Instability and decay of the primary structure of DNA. Nature.

[CR31] Lleò MM, Pierobon S, Tafi MC, Signoretto C, Canepari P (2000). mRNA detection by reverse transcription-PCR for monitoring viability over time in an *Enterococcus faecalis* viable but nonculturable population maintained in a laboratory microcosm. Appl Environ Microbiol.

[CR32] Lundgren M, Malandrin L, Eriksson S, Huber H, Bernander R (2008). Cell cycle characteristics of *Crenarchaeota*: unity among diversity. J Bacteriol.

[CR33] Makarova KS, Aravind L, Wolf YI, Tatusov RL, Minton KM, Koonin EV (2001). Genome of the extremely radiation-resistant bacterium *Deinococcus radiodurans* viewed from the perspective of comparative genomics. Microbiol Mol Biol Rev.

[CR34] Mao D, Grogan DW (2017). How a genetically stable extremophile evolves: Modes of genome diversification in the archaeon *Sulfolobus acidocaldarius*. J Bacteriol.

[CR35] Margulis L (1976). Genetic and evolutionary consequences of symbiosis. Exp Parasitol.

[CR36] Mattimore V, Battista JR (1996). Radioresistance of *Deinococcus radiodurans*: functions necessary to survive ionizing radiation are also necessary to survive prolonged desiccation. J Bacteriol.

[CR37] Miller SL, Lazcano A (1995). The origin of life- did it occur at high temperature?. J Mol Evol.

[CR38] Moeller R, Raguse M, Leuko S, Berger T, Hellweg CE, Fujimori A (2017). STARLIFE—an international campaign to study the role of galactic cosmic radiation in astrobiological model systems. Astrobiology.

[CR39] Moissl-Eichinger C (2011). Archaea in artificial environments: their presence in global spacecraft clean rooms and impact on planetary protection. ISME J.

[CR40] Nisbet EG, Sleep NH (2001). The habitat and nature of early life. Nature.

[CR41] Oliver JD, Colwell RR, Grimes DJ (2000). The public health significance of viable but nonculturable bacteria. Nonculturable microorganisms in the environment.

[CR42] Pace NR (1991). Origin of life - facing up to the physical setting. Cell.

[CR43] Paper W, Jahn U, Hohn MJ, Kronner M, Näther DJ, Burghardt T (2007). *Ignicoccus hospitalis* sp. nov., the host of ‘*Nanoarchaeum equitans*’. Int J Syst Evol Microbiol.

[CR44] Podar M, Anderson I, Makarova KS, Elkins JG, Ivanova N, Wall MA (2008). A genomic analysis of the archaeal system Ignicoccus hospitalis-*Nanoarchaeum equitans*. Genome Biol.

[CR45] Saran M, Bors W (1997). Radiation chemistry of physiological saline reinvestigated: evidence that chloride-derived intermediates play a key role in cytotoxicity. Radiat Res.

[CR46] Shuryak I (2019). Review of microbial resistance to chronic ionizing radiation exposure under environmental conditions. J Environ Radioact.

[CR47] Spaans SK, van der Oost J, Kengen SWM (2015). The chromosome copy number of the hyperthermophilic archaeon *Thermococcus kodakarensis* KOD1. Extremophiles.

[CR48] Tillett D, Neilan BA (2000). Xanthogenate nucleic acid isolation from cultured and environmental cyanobacteria. J Phycol.

[CR49] White MF, Allers T (2018). DNA repair in archaea - an emerging picture. FEMS Microbiol Rev.

[CR50] Whitesides MD, Oliver JD (1997). Resuscitation of *Vibrio vulnificus* from the viable but nonculturable state. Appl Environ Microbiol.

[CR51] Xu H-S, Roberts N, Singleton FL, Attwell RW, Grimes DJ (1982). Survival and viability of nonculturable *Escherichia coli* and *Vibrio cholera* in the estuarine and marine environment. Microb Ecol.

